# Diel patterns of predation and fledging at nests of four species of grassland songbirds

**DOI:** 10.1002/ece3.7541

**Published:** 2021-05-01

**Authors:** Christine A. Ribic, David J. Rugg, Kevin Ellison, Nicola Koper, Pamela J. Pietz

**Affiliations:** ^1^ U.S. Geological Survey, Wisconsin Cooperative Wildlife Research Unit University of Wisconsin Madison WI USA; ^2^ U.S. Forest Service, Research and Development Madison WI USA; ^3^ Northern Great Plains Program American Bird Conservancy Bozeman MT USA; ^4^ Natural Resources Institute University of Manitoba Winnipeg MB Canada; ^5^ U.S. Geological Survey (emeritus) Northern Prairie Wildlife Research Center Jamestown ND USA

**Keywords:** diel predation activity, fledging, grassland birds, nest predation

## Abstract

Although it is common for nestlings to exhibit a strong bias for fledging in the morning, the mechanisms underlying this behavior are not well understood. Avoiding predation risk has been proposed as a likely mechanism by a number of researchers. We used video surveillance records from studies of grassland birds nesting in North Dakota, Minnesota, and Wisconsin to determine the diel pattern of nest predation and fledging patterns of four ground‐nesting obligate grassland passerines (Grasshopper Sparrow (*Ammodramus savannarum*), Savannah Sparrow (*Passerculus sandwichensis*), Bobolink (*Dolichonyx oryzivorus*), and Eastern Meadowlark (*Sturnella magna*)). We used the nest predation pattern as a surrogate for predation activity to test whether nestlings minimized predation risk by avoiding fledging when predation activity was high and preferentially fledging when predation risk was low. Predation activity was significantly lower starting 3 hr before sunrise and ending 3 hr after sunrise, followed by a transition to a period of significantly higher activity lasting for 4 hr, before declining to an average activity level for the rest of the diel period. There was little evidence that the four grassland bird species avoided fledging during the high‐risk period and Savannah Sparrow fledged at higher rates during that period. All four species had hours during the low‐risk period where they fledged at higher rates, but only Grasshopper Sparrow fledged preferentially during that period. Bobolink and Eastern Meadowlark had multiple hours with high fledging rates throughout the daytime period, resulting in no relationship between probability of fledging and predation risk. Given the species variability in fledging pattern seen in our study, it is unlikely that there is a universal response to any driver that affects time of fledging. Further study is needed to understand the complex interplay between species ecology and drivers such as physiology, energetics, and predation in affecting grassland bird fledging behavior.

## Graphical abstract

We investigated the predation activity pattern affecting four obligate grassland bird species and whether that affected the time of fledging. Nest predators had the lowest activity 3 hr before and after sunrise (blue bar) with their highest activity during mid‐morning to early afternoon (red bar). Overall, while the birds fledged significantly more during certain hours across the morning, there was little evidence that predation risk was a driver affecting the species' fledging patterns.

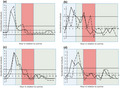

## 1 INTRODUCTION

Ground‐nesting grassland bird species tend to fledge in the morning (Pietz, Granfors, & Grant, 2012; Ribic, Ng, et al., 2018), a behavior also observed in many other songbirds (e.g., Chiavacci et al., 2015; Johnson et al., 2004; Lemel, 1989; Skutch, 1953). However, why nestlings of grassland birds exhibit this behavior is not well understood. The days following fledging are known to constitute a period of high predation mortality for fledglings (see reviews by Cox et al., 2014 and Naef‐Daenzer & Grüebler, 2016). Thus, a common thought has been that fledging early is a predation risk avoidance strategy, and it acts by providing the new fledgling enough time to find a safe harbor before nightfall (Perrins 1979). As noted by Chiavacci et al. (2015), who labeled this version of predation risk avoidance the “maximum time hypothesis,” this approach implies that night is a period of increased predation risk. While nocturnal predation does occur on grassland bird nests, the major predators of grassland bird nests are diurnal (Pietz, Granfors, & Ribic, 2012), so it was not unexpected that Ribic, Ng, et al. (2018) did not find support for the predation risk hypothesis. However, as Ribic, Ng, et al. (2018) and Ribic et al. (2019) observed, predation may still shape grassland bird nesting ecology in other ways. For example, if grassland birds are able to determine relative predation risk (Ibanez‐Alamo et al., 2015; Weatherhead & Blouin‐Demers, 2004) and the relative pattern of risk is consistent over time, a result of evolution might be a pattern of preferentially fledging during times when predation risk was relatively low and avoiding fledging during times when predation risk was relatively high.

Predation risk at the time of fledging integrates two separate processes. The first process is encounter risk, that is, the risk that a nestling, upon leaving the nest, will encounter a predator. This risk can vary over the course of a day as the predators active on the landscape vary, which depends on the different predators' diel cycles. The second process is encounter response risk, that is, the risk that a fledgling's response to an encounter with a predator will fail to result in the fledgling escaping the predator. When encounter response risk is high, such as at the time of fledging when nestling locomotory ability is not well‐developed (Heers, 2018; Jones et al., 2017; Ribic et al., 2019), we hypothesize that lowering predation risk becomes more dependent on reducing encounter risk and thus that fledging would occur during periods when encounter risk is lower. Therefore, a better understanding of predation pressures faced by fledglings in grassland ecosystems, and how those pressures change over the course of a day, would allow us to conduct a more robust assessment of the influence of predation risk.

Grassland passerine nests are depredated by a diverse community composed of species that span a broad range of sizes (e.g., mice to deer), taxa (e.g., birds, snakes, mammals), and general diel activity (e.g., nocturnal, crepuscular, diurnal) (Pietz, Granfors, & Ribic, 2012). However, there is limited information about the diel activity patterns of those predator species beyond their general patterns (Cornell Lab of Ornithology, 2020; Ernst & Ernst, 2003; Feldhamer et al., 2003). This reflects a general paucity of information in the literature on the diel pattern of predation risk (Gill et al., 2016). Predation activity at grassland bird nests provides a good index of the diel activity pattern of the predator species outside the nest (i.e., the encounter risk pattern) because predation at grassland bird nests is opportunistic (Vickery et al., 1992).

The development of video surveillance systems for use at nests in grasslands (Ribic, Thompson, & Pietz, 2012) allows us to begin to investigate whether there is a discernible relationship between predation activity and fledging activity. Therefore, our objectives were to (a) determine the diel pattern of grassland bird nest predation, including determining the major species or species groups that contribute to the pattern, (b) construct a diel pattern of classified relative nest predation risk (high, average, low periods) to serve as a proxy for the pattern of fledgling predator encounter risk, (c) compare the pattern of the time of grassland bird fledging (by species and order of fledging) to the pattern of classified predator encounter risk, and (d) determine whether predator encounter risk plays a significant role in time of fledging as reflected in under‐use of periods of high risk or over‐use of periods of low risk relative to availability of those periods. The order of fledging comparison is of interest because Ribic, Ng, et al. (2018), found significant differences in diel patterns of fledging between first nestlings to fledge and the subsequent nestlings to fledge, and it is therefore possible that these two groups use different fledging time strategies.

## 2 MATERIALS AND METHODS

### 2.1 Study sites

We used a subset of video records from the published grassland bird nesting studies used by Ribic, Ng, et al. (2018). Specifically, we used video records from study sites located in native mixed‐grass and remnant tall‐grass prairie and warm‐season and cool‐season fields enrolled in the U.S. Department of Agriculture's Conservation Reserve Program (CRP). The prairie sites were located in North Dakota, USA near Jamestown (46.9000°N, 98.7167°W), Woodworth (47.1333°N, 99.3000°W), and Upham (48.5833°N, 100.7333°W) and in Minnesota, USA near Crookston (47.7833°N, 96.6167°W). The warm‐ and cool‐season grass fields were in Wisconsin, USA near Mt. Horeb (43.0167°N, 89.7500°W). Further information on the studies is in Ribic, Ng, et al. (2018).

### 2.2 Predation times

We collated the time of all nest predation events (partial or complete nest predations, forced fledging events, scavenging of unhatched eggs, or dead nestlings) from previous studies, combining data across predator species to develop the diel pattern of predation risk (Byers et al., 2017; Ellison et al., 2013; Pietz, Granfors, & Ribic, 2012; Ribic, Guzy, et al., 2012). Nest scavenging events were included because predators encounter nests, whether active or inactive, opportunistically (Vickery et al., 1992), and thus, a nest scavenging event is just another predator encounter in the overall measure of encounter risk. Following Ribic, Ng, et al. (2018), we adjusted for latitudinal differences in sunrise across the studies by translating predation times to time relative to local sunrise in decimal hours. Times of local sunrise were determined from the U.S. Naval Observatory (2016). Local sunrise was used to define the new day, so negative times were possible (i.e., predation could occur after local midnight but before local sunrise).

All predation events for which the predator could be identified were categorized into mammal, bird, or reptile (i.e., snake) groups. The groups were further split into nocturnal, crepuscular, and diurnal activity categories using information in Feldhamer et al. (2003) for mammals, Ernst and Ernst (2003) for reptiles, and Cornell Lab of Ornithology (2020) for birds. If the predator could not be identified, the predation event was categorized as unknown. All scientific names were obtained from the Integrated Taxonomic Information System (https://www.itis.gov).

### 2.3 Fledging times

Fledging time in relation to sunrise was measured for all fledglings by Ribic, Ng, et al. (2018). We used only data from obligate grassland bird nests (Vickery et al., 1999) because the impact of evolutionary pressures from predation should be most apparent in those species that require grasslands for reproduction; generalist and facultative grassland birds are presumably subject to potentially different predation regimes depending on the habitat within which the nest was located, making it more difficult to have a consistent pattern to react to. We used the obligate grassland birds with the largest sample sizes for fledging; these were Bobolink (*Dolichonyx oryzivorus*) (149 fledging events from 51 nests), Eastern Meadowlark (*Sturnella magna*) (128 events from 46 nests), Savannah Sparrow (*Passerculus sandwichensis*) (62 events from 21 nests), and Grasshopper Sparrow (*Ammodramus savannarum*) (53 events from 19 nests). These species also provided a range of average times of first fledging with Grasshopper Sparrow starting the earliest in the morning (3.75 hr after sunrise), followed by Savannah Sparrow and Bobolink (4.1 hr), and then Eastern Meadowlark (5.07 hr) (Ribic, Ng, et al., 2018).

### 2.4 Analysis

#### 2.4.1 Comparison of nest predation temporal patterns across habitats

Fledging patterns of obligate grassland birds differed before and after the solstice (Ribic, Ng, et al., 2018) and a change in predation activity pattern might explain this. Therefore, we determined whether there were pre‐ and postsolstice differences in predation activity patterns within prairie, cool‐season CRP fields, and warm‐season CRP fields. After that analysis, we assessed whether there were differences in diel predation patterns across prairie, cool‐season CRP fields, and warm‐season CRP fields to determine whether the predation patterns differed by type of grass habitat. We were not interested in yearly variation because we were looking for signals that had enough consistency across space and time to create a selection pressure. We tested whether the predation time cumulative distribution functions came from the appropriate common continuous distribution that is otherwise unspecified. We used the nonparametric k‐sample Anderson‐Darling (AD) test, which is a generalization of the 2‐sample Kolmogorov–Smirnov test (Scholz & Stephens, 1987). We used R version 4.0.0 (R Core Team, 2020) and the R package *kSamples* to run the AD test with method = “simulated” (10,000 simulations). We used the results from AD Test 2, which models the tails of the distribution better than Test 1 (Scholz & Stephens, 1987). Significance was assessed at *α* of 0.05.

#### 2.4.2 Defining diel predation activity

Using the results of the nest predation pattern analyses, we appropriately combined depredation time data (i.e., where the AD tests did not reject the null hypotheses of no season and habitat differences). To determine if the nest predation pattern varied across the diel cycle, we used a contingency table approach with residual analysis (Fisher, 1993; Lloyd, 1999). This allowed us to test the diel pattern against a uniform distribution and provided information about lack of model fit that we could use to define a general diel predation activity pattern. We grouped nest predation times into hour bins and tested whether the binned nest predation pattern followed a uniform distribution using TableSim (Rugg, 2003) with 20,000 simulations. Significance was assessed at α of 0.05. Because we had no a priori information about what the diel predation activity pattern should be, we defined a general pattern of high, average, and low nest predation activity across the diel cycle from the standardized residuals. These periods were considered an index of relative predator encounter risk in the grassland habitat.

Under the null hypothesis, standardized residuals follow a Normal (0,1) distribution (Lloyd, 1999), so we used values of the Normal (0,1) distribution for the upper and lower 5% tails of the distribution to categorize hours into high and low predation activity, respectively, with the remaining hours being average predation activity. Specifically, hours with residuals ≥1.69 were categorized as having high predation activity, hours with residuals ≤ −1.69 were categorized as having low predation activity, and those hours with residuals between −1.69 and 1.69 were categorized as having average predation activity. We then grouped the hours into relative predation activity periods, and we chose to require that a period had to be at least 2 hr in length. We defined a period of high predator encounter risk to be a run of hours that started and ended with an hour in the high predation activity category and could include single hours with a different activity category (i.e., a single hour with a different activity category would not break the run). A period of low predator encounter risk was a run of hours that started and ended with an hour in the low predation activity category. Periods of average predator encounter risk were runs of hours in the average predation activity category.

We calculated the hourly relative predator encounter risk for each period by dividing the total number of depredations that occurred in a period by the total number of hours in that period. We also used the hourly values to determine the relative changes in predation encounter risk between average risk and high and low risk. We plotted the standardized residuals against hour, with relative predator encounter risk periods displayed on the graph for comparison. We report the fraction of the standardized residuals that fell within the nominal 5% tails.

We calculated the proportion of total nest predations by predator group to understand what predator species groups contributed to the diel nest predation pattern. We then ordered the proportions and focused on the groups whose summed proportions made up at least 90% of the total nest predations. To illustrate when the different predator groups were active, we computed an individual group's hourly nest predations in relation to the total nest predations for that group and overlaid those hourly proportions on the diel nest predation activity pattern defined from total nest predations (from above). Total nest depredations by predator species are presented in Appendix 1.

#### 2.4.3 Fledging time pattern versus predation activity pattern

While the results of Ribic, Ng, et al. (2018) imply that nestlings do not fledge at random throughout the daytime period, we formally tested this for each species. If the null hypothesis of fledging at random was rejected for a species, we then used the daytime predator activity pattern as a mechanism‐based theoretical distribution that might explain the fledging pattern. To determine if nestlings were fledging at times to minimize relative predator encounter risk, we compared the pattern of grassland bird fledging times to the pattern of predation activity at two temporal scales. The first was a fine‐scale analysis that compared the diel pattern of fledging events by hour against the diel pattern of predation activity by hour. These analyses provided detailed information on variation in how the fledging patterns overlapped with the predation activity periods. The second approach was a coarse‐scale analysis used to determine how completely the bird species' patterns of fledging coincided with the three predation activity periods. In this case, we were interested in the proportions of fledging events that occurred in the three predation activity periods, regardless of temporal pattern.

Grassland birds fledged almost exclusively during the diurnal period of the diel cycle (Ribic, Ng, et al., 2018). Therefore, to compare the individual species' fledging time patterns against the diel predator encounter risk pattern, we restricted the analysis to a time period when the nestlings could fledge. Grassland birds are awake in the nest prior to dawn (during civil twilight) and are active for up to a half hour after sunset when the parent returns to the nest for the night (Slay et al., 2012), so nestlings could theoretically fledge anytime during that time period. Therefore, we compared the fledging and predation activity patterns in the 17‐hr period starting with 1 hr before dawn (contains civil twilight) through 15 hr after dawn (contains sunset); we refer to this time period as the daytime period. For the fine‐scale analyses, the predation activity hours that fell within the daytime period constituted the daytime predation activity pattern and we grouped the fledging times into the daytime hour bins for an hourly daytime fledging pattern.

To test the null hypothesis that fledging took place at random, we had to determine an appropriate distribution for the null model. This entailed thinking about how to model a nestling's decision to fledge. With our data binned into hours, the common comparison to a uniform distribution required nestlings to decide their hour of fledging by throwing a fair, 17‐sided die at the start of the day. This seemed to be an unlikely decision process for a null model. Instead, we modeled the decision to fledge as a series of independent choices made every hour to either fledge (success) or not (failure) with some constant probability of fledging (*p*). This defines the geometric distribution (Lindgren, 1976). We computed the probability that an individual fledged at hour *k* from the standard formula: P(hour of fledging = *k*) = (1 − *p*)^(^ *^k^* ^− 1)^ *p*, where *k* = 1,…, 17 (the number of hours in the daytime period). We estimated p for each species using the average of the hourly proportions of fledging. We tested the null hypothesis that the species fledging time pattern followed this random pattern using the “theory” option in TableSim and 20,000 simulations; significance was assessed at *α* of 0.05.

We followed a similar analysis approach for the predation activity pattern. We tested the null hypothesis that fledging times followed the daytime predation activity pattern using the “theory” option in TableSim and 20,000 simulations and assessed significance at *α* of 0.05; the theoretical probability distribution was the hourly predation activity (see above for how this was calculated). We then used the sets of standardized residuals to give us information about how the fledging patterns differed from the daytime predation activity pattern. We identified hours when nestlings fledged significantly less than expected and hours when fledging occurred significantly more than expected using residuals significant at *α* of 0.10. We used this significance level to better capture any trends in the pattern of deviations (i.e., we are giving the hypothesis of predator encounter risk avoidance a better opportunity to be supported). We were interested in whether those hours coincided with the predation activity periods (e.g., significantly positive residuals primarily found in the low predation activity period, not spread across the periods). The standardized residuals for the tests by species and fledging order are presented in Appendix 2.

For the coarse‐scale analyses, we focused on proportions of fledging events that occurred in the three predation activity periods. We first tested for differences between fledging groups using a 2 × 3 contingency table for each species (first and subsequent fledging groups and three predation activity periods). The null hypothesis was that the distribution of fledging events across predation activity periods was the same for both fledging groups. If the first and subsequent fledging group patterns were the same, we combined the data by species and then tested whether the proportions of fledging in the predation activity periods were the same across species. We tested the contingency tables with TableSim (Rugg, 2003), using the “strata” option for comparing across fledging groups and the “marginal” option for comparing across species. We used 20,000 simulations for each test and significance was assessed at *α* of 0.05. If the test was significant, we used residual analysis to determine where differences in proportions occurred; residuals ≥1.96 indicated where significantly more fledging occurred in relation to a specific predation activity period.

Our second coarse‐scale test assessed uniformity with respect to the predator activity periods. We did this by expanding the coarse‐scale comparisons described above to evaluate whether species' fledging activity was random with respect to the three predation activity periods (i.e., used in proportion to availability). For these tests, we used the “theory” option in TableSim, with the theoretical probability distribution being the fraction of daytime hours classified into each of the three predation activity periods. As before, we used 20,000 simulations, and significance was assessed at *α* of 0.05. If the test was significant, we used residual analysis to determine where differences in proportions occurred; residuals ≥1.96 indicated significantly more fledging occurred in relation a specific predation activity period.

Nest predations and fledging times categorized by hour are presented in Appendix 3.

## 3 RESULTS

### 3.1 Diel predation activity pattern

There were 392 depredation events documented across the three grassland habitats: 172, 107, and 113 events in cool‐season CRP fields, warm‐season CRP fields, and prairie, respectively. Diel predation patterns pre‐ and postsolstice in the habitats did not differ (AD = 6.66, simulated *p* = .16); patterns across the habitats also did not differ (AD = 2.71, simulated *p* = .20). Therefore, analysis proceeded on the fully combined data set.

**FIGURE 1:**
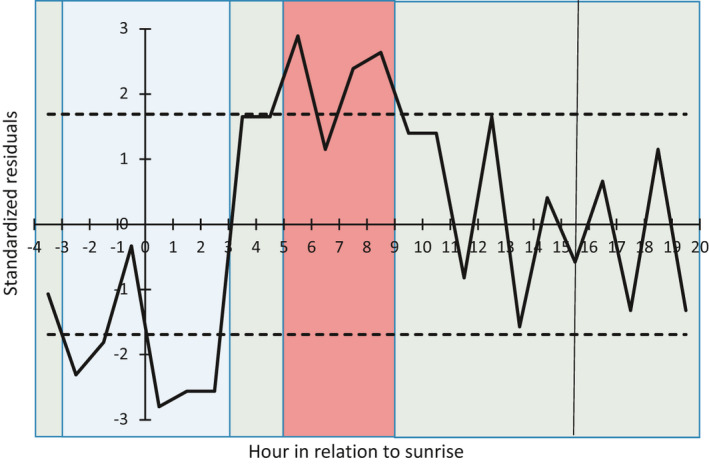
Standardized residuals from the test of predation activity having a uniform distribution across the diel period plotted against hour in relation to sunrise. The solid black line connects the hourly residuals to show the pattern of deviation from the uniform. Positive residuals indicate more predation activity than expected under the uniform distribution; negative residuals indicate less predation activity than expected under the uniform distribution. The solid vertical line in the hour 15 bin indicates the hour bin in which local sunset occurred. Local sunrise is hour 0. The dotted lines at ±1.69 are the cutoff points used to define periods of low, average, and high predation activity. Blue coloring indicates periods of relatively low predation activity (residuals < −1.69), green coloring indicates periods of average predation activity (residuals between −1.69 and 1.69), and red coloring indicates periods with relatively high predation activity (residuals >1.69)

Nest predation was not uniform over the 24‐hr cycle (*χ* ^2^ = 85.8, *p* < .0001). There was one period of relatively high predation activity from 5 hr after sunrise to 9 hr after sunrise (late morning to early afternoon) (Figure 1) (17% of the diel cycle). There was one period of relatively low predation activity starting 3 hr before dawn and extending into the early morning to 3 hr after dawn (Figure 1) (25% of the diel cycle). The morning period (from 3–5 hr after dawn) when predation activity was at average levels reflected a transition between the high and low periods (Figure 1). Otherwise, predation activity was at the average level for an extended period in the afternoon and early evening (Figure 1).

Over the 24‐hr diel cycle, average predation activity occurred across 14 hr, which captured 62% of nest depredations. Therefore, an hour during the average activity period captured 4.4% of nest depredations. The 4 hr that comprised the high predation activity period captured 26% of nest depredations, for an average hourly level of 6.5% of depredations (47% higher than during the average predation period). In contrast, the 6 hr that comprised the low predation activity period captured 12% of nest depredations, for an average hourly level of 2.0% of depredations (54% lower than during the average predation period).

**FIGURE 2:**
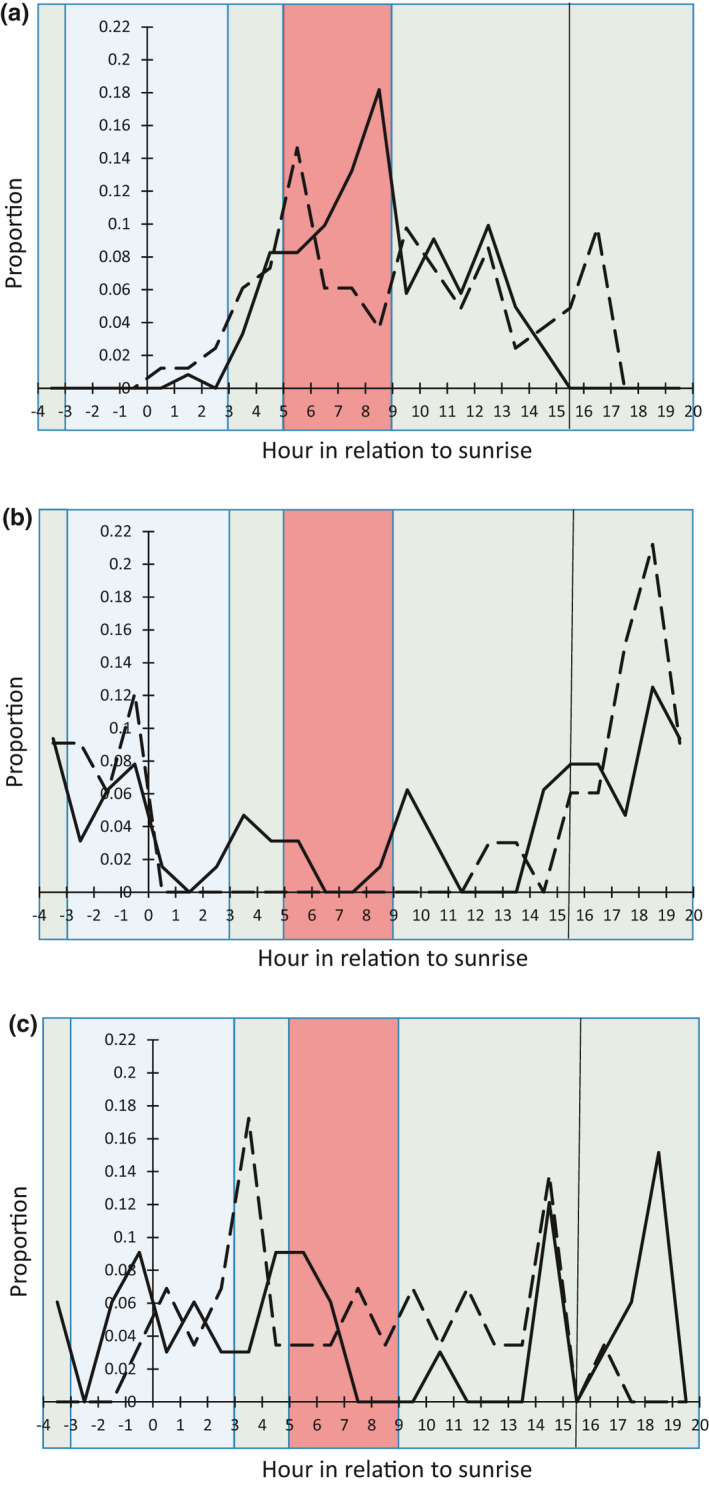
Hourly proportion of nest depredations by (a) ground squirrels (solid line) and snakes (dashed line), (b) grassland‐associated meso‐mammals (solid line) and forest‐associated meso‐mammals (dashed line), and (c) mice/voles (solid line) and birds (dashed line). Proportions are in relation to the total predations for the individual group. The solid vertical line in the hour 15 bin indicates the hour bin in which local sunset occurred. Local sunrise is hour 0. Blue coloring indicates periods of relatively low predation activity, green coloring indicates periods with average predation activity, and red coloring indicates periods with relatively high predation activity

Three predator groups depredated about two‐thirds of the nests: ground squirrels (95.9% of ground squirrel nest depredations were thirteen‐line ground squirrel [*Ictidomys tridecemlineatus*]), snakes (primarily diurnal western foxsnake [*Pantherophis ramspotti*] with 56.1% of snake depredations and nocturnal eastern milksnake [*Lampropeltis triangulum*] with 29.3% of snake depredations), and grassland‐associated meso‐mammals (primarily striped skunk [*Mephitis mephitis*] with 50.8% of meso‐mammal depredations) (Table 1). The major species groups that were active during the daylight hours of high predation activity were ground squirrels and snakes, with ground squirrel activity increasing toward the late morning/early afternoon (Figure 2a). The major species groups active during hours of low predation activity prior to sunrise were forest‐associated and grassland‐associated meso‐mammals (Figure 2b). Mice/voles and birds predated nests at low levels throughout the diel period (Figure 2c).

**TABLE 1:** Percentages of 392 grassland songbird nest depredations by major predator groups in prairie and warm‐season and cool‐season Conservation Reserve Program fields

Predator Group	Active period	% total nest depredations
Ground squirrels	Diurnal	30.9
Snakes	Diurnal/nocturnal	20.9
Meso‐mammals (grassland‐associated)	Diurnal/crepuscular/nocturnal	16.3
Meso‐mammals and large mammals (forest‐associated)	Crepuscular/nocturnal	8.4
Mice/voles	Diurnal/nocturnal	8.4
Birds	Diurnal	7.4

Note

These groups made up 92.3% of the total nest depredations.

Although the 17‐hr daytime period when nestlings fledge constituted 70.8% of available time, 77.0% (302) of the nest predation events occurred during this period. Nine of the 14 hr in the average predation activity period occurred in the daytime and captured 55.6% of daytime nest depredations. Therefore, an hour of average predation activity during daytime captured 6.2% of daytime nest depredations. The high predation activity period (4 hr) only occurred during daytime and captured 33.8% of daytime nest depredations, for an average hourly level of 8.4% (37% higher than average). Four of the 6 hr of low predation activity occurred during daytime and captured 10.6% of daytime nest depredations, for an average hourly level of 2.6% (57% lower than average).

### 3.2 Fledging pattern in relation to relative predation activity in grassland habitat

#### 3.2.1 Daytime pattern comparisons (fine‐scale)

No species fledged at random (Grasshopper Sparrow: *χ* ^2^ = 67.7, *p* < .001; Savannah Sparrow: *χ* ^2^ = 43.57, *p* = .001; Bobolink: *χ* ^2^ = 128.6, *p* < .001; Eastern Meadowlark: *χ* ^2^ = 126.0, *p* < .001). With fledging at random rejected for all species, we proceeded to compare fledging patterns against the daytime predation activity pattern for each of the four grassland bird species.

**FIGURE 3:**
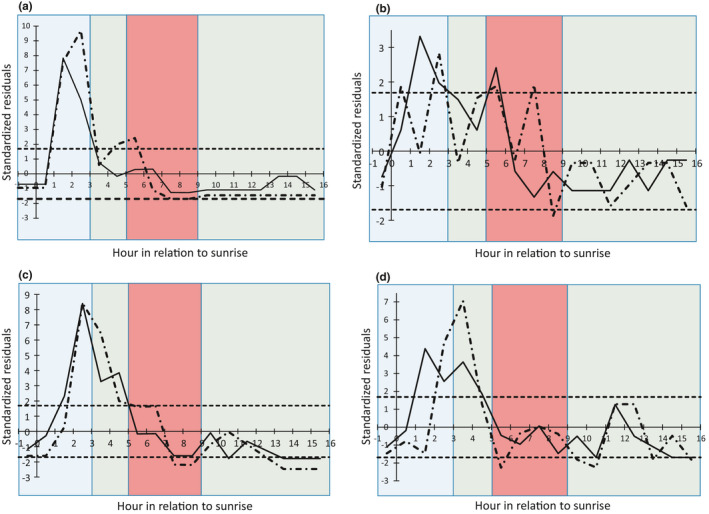
Standardized residuals from the test of the fledging pattern of (a) Grasshopper Sparrow, (b) Savannah Sparrow, (c) Bobolink, and (d) Eastern Meadowlark being the same as the pattern of relative predation activity during the daytime (civil twilight through sunset) plotted against hour in relation to sunrise. The solid black line is the pattern of residuals from the fledging pattern of the first nestlings to leave; the dotted and dashed black line is the pattern of residuals from the fledging pattern of the nestlings that left afterward. Positive residuals indicate more fledging activity than expected relative to the rate of predation activity; negative residuals indicate less fledging activity than expected relative to the rate of predation activity. Local sunrise is hour 0. The dotted lines at ±1.69 represent residuals significant at α of 0.10. Blue coloring indicates periods of relatively low predation activity, green coloring indicates periods with average predation activity, and red coloring indicates periods with relatively high predation activity

For Grasshopper Sparrow, the hourly patterns for first fledging events and subsequent fledging events were significantly different from the hourly pattern for relative daytime predation activity (first fledge: *χ* ^2^ = 97.2, *p* < .0001; subsequent fledges: *χ* ^2^ = 184.9, *p* < .0001). There were significantly more first and subsequent fledging events during two of the 4 hr in the morning period of low predation activity (Figure 3a). The hourly rate of fledging for Grasshopper Sparrow during the period of low predation activity was 13.2%, regardless of fledging order. Additional subsequent fledging events occurred more during the last hour of the morning period of average predation activity and the first hour of the high predation activity period (Figure 3a). There were significantly fewer subsequent fledging events in the last 2 hr in the high predation activity period (Figure 3a). The hourly rate of fledging for Grasshopper Sparrow during the period of high predation activity was 5.7%.

For Savannah Sparrow, the patterns for first fledging events and subsequent fledging events were significantly different from the pattern for relative daytime predation activity (first fledge: *χ* ^2^ = 32.1, *p* = .016; subsequent fledges: *χ* ^2^ = 32.4, *p* = .01). There were significantly more first fledging events during 2 hr of the morning period of low predation activity; however, there were also significantly more first fledging events during the first hour of the morning period of high predation activity (Figure 3b). The hourly rate of fledging for the first Savannah Sparrows to leave the nest during the period of low predation activity was 7.1%. However, the hourly rate of fledging for the first Savannah Sparrows to leave the nest during the period of high predation activity was 8.3%. Subsequent fledging events followed a more distributed pattern, with more fledging occurring in 2 hr of the morning low predation activity period and 2 hr of the morning high predation activity period; significantly less fledging occurred during the last hour of the high predation activity period (Figure 3b).

For Bobolink, the patterns for first fledging events and subsequent fledging events were significantly different from the pattern for relative predation activity (first fledge: *χ* ^2^ = 122.6, *p* < .0001; subsequent fledges: *χ* ^2^ = 159.2, *p* < .0001). There were significantly more first fledging events during the last 2 hr of the morning period of low predation activity; this carried over into the 2‐hr morning period of average predation activity (Figure 3c). Subsequent fledging events had a similar pattern for the morning low and average predation activity periods (Figure 3c). In addition, there were significantly fewer subsequent fledging events during the last 2 hr of the high predation activity period and fewer first and subsequent fledging events during 3 hr of the afternoon average predation activity period near sunset (Figure 3c). The hourly rate of fledging for Bobolink during the period of low predation activity was 5.9%, regardless of fledging order. The hourly rate of fledging for Bobolink during the period of high predation activity was 6.7%, regardless of fledging order.

For Eastern Meadowlark, the pattern for first fledging events and subsequent fledging events were significantly different from the pattern for relative predation activity (first fledge: *χ* ^2^ = 59.0, *p* < .0001; subsequent fledges: *χ* ^2^ = 102.3, *p* < .0001). There were significantly more first fledging events during the last 2 hr of the morning period of low predation activity; this carried over into the 2‐hr morning period of average predation activity. There were significantly fewer fledging events that occurred in 3 hr of the afternoon average predation activity (Figure 3d). The patterns of the subsequent fledging events were similar, with the hours of increased subsequent fledging events shifted later in the morning; significantly fewer fledging events occurred in 4 hr of the afternoon average predation activity (Figure 3d). The hourly rate of fledging for Eastern Meadowlark during the period of low predation activity was 6.0%, regardless of fledging order. The hourly rate of fledging for Eastern Meadowlark during the period of high predation activity was 3.9%.

#### 3.2.2 Fledging events in relation to predation activity periods (coarse‐scale)

The proportion of fledging events that occurred during the three predation activity periods did not differ between the nestlings who fledged first and those who left afterward (*χ* ^2^ = 7.5, *p* > .75) for any of the species, consistent with what can be inferred from the fine‐scale analyses. Therefore, the two types of fledging events were combined for species comparisons. Species differed significantly in how fledging events were distributed across the predation activity periods (*χ* ^2^ = 35.8, *p* < .0001). Grasshopper Sparrow was significantly different from the other three species with a significantly high residual in the low predation activity period (residual = 4.0) and a significantly low residual in the average predation activity period (residual = −2.5).

Proportions of fledging events that should occur during the three predation activity periods if the periods were being used according to availability were 23.5% for the low and high predation activity periods and 53% for the average predation activity period. Grasshopper Sparrow and Savannah Sparrow nestlings did not fledge at the rates based on availability (Grasshopper Sparrow: *χ* ^2^ = 27.5, *p* < .0001; Savannah Sparrow: *χ* ^2^ = 8.5, *p* < .015). Grasshopper Sparrow nestlings fledged at a higher rate (52.8%) during the low predation activity period (residual = 4.4), fledged at a lower rate in the average period (24.5%) (residual = −2.9), and fledged during the high period in proportion to availability (22.6%) (residual = −0.13). Savannah Sparrow nestlings fledged at a higher rate during the high period (38.7%) (residual = 2.5), fledged at a slightly lower rate during the average period (38.7%) (residual = −1.6), and fledged during the low period in proportion to availability (22.5%) (residual = −0.15). In contrast, Bobolink and Eastern Meadowlark nestlings fledged at rates in proportion to availability of the predation activity periods (Bobolink: *χ* ^2^ = 1.0, *p* > .55; Eastern Meadowlark: *χ* ^2^ = 4.0, *p* = .15). The rates of fledging for Bobolink in the three periods were 26.8% during the high period, 23.5% during the low period, and 49.7% during the average period. Rates of fledging for Eastern Meadowlark in the periods were 15.6% for the high period, 24.2% for the low period, and 60.2% for the average period.

## 4 DISCUSSION

We found that predator encounter risk in grassland habitats, as reflected by nest predation patterns, was consistent over the course of the season and the grassland types in which our data were collected. In addition, the pattern was not uniform across the diel cycle. There were two periods of lower and higher predation activity separated by two periods of average activity, with the higher predation activity period spanning mid‐morning to early afternoon.

Our finding that the period of highest predation activity occurred during the diurnal period of the diel cycle is consistent with the finding of Davis et al. (2012) for Sprague's Pipit (*Anthus* *spragueii*). Our findings of highest predation activity during the diurnal period and lower predation activity in the hours after sunrise are qualitatively similar to the findings of Gill et al. (2016) for forest birds, despite differences in patterns of nest predation and predator community composition between the forest and grassland systems. In our study, the period of significantly lower predation activity was centered around dawn. There were few nocturnal mammals active during the low period prior to sunrise. The continued lower predation activity into the early morning (after sunrise) appeared to be a consequence of reduced activity by nocturnal mammals and a lag in the start of diurnal predation activity, specifically the activity of the two main diurnal predators, thirteen‐lined ground squirrel and western foxsnake.

Thirteen‐lined ground squirrel activity is dependent on thermal conditions (Vispo & Bakken, 1993) with morning emergence affected by dew point (McCarley, 1966). McCarley (1966) found that thirteen‐lined ground squirrels became active around 0700 local time in May and June, but emergence was delayed to later in the morning (1,000 local time) when there was heavy dew. Effects of temperature and changes in activity due to precipitation (in general) on activity have been found in other ground squirrel species (e.g., Long et al., 2005; Shaw, 1945; Vaczi et al., 2006). Likely other predator species at higher trophic levels also contributed to the temporal activity patterns for ground squirrels but more research would be needed to understand these complex interactions.

A review of preferred body temperature among 55 snake species from five families (Lillywhite, 1987) found that the majority had mean preferred body temperatures between 28 and 34°C, with most close to 30°C. While snake activity often reflects temperature constraints (Blouin‐Demers and Weatherhead 2001, Ernst & Ernst, 2003; George et al., 2015), detailed information on diel activity among the specific species depredating grassland bird nests in our system is limited—we found one reference to western foxsnake actively searching for prey in the morning (0600–1100 local time) and evening (1600–2000 local time) (Ernst & Ernst, 2003). In other systems, snakes reduced activity after sunrise (0600–1000; see Blouin‐Demers and Weatherhead 2001).

The predator species' activity patterns, then, created a window of lower predator activity in the early morning, when neither nocturnal nor diurnal predators were relatively active. The differences in the activity of ground squirrels and snakes were reflected in the patterns in nest predation in our study. Depredations by snakes started earlier than those by thirteen‐lined ground squirrel but both were highest starting a few hours after dawn, resulting in the diurnal period of high predator activity. However, predator communities may vary on large geographic scales (e.g., Thompson & Ribic, 2012), which may result in different predation activity patterns.

We know of no other studies that have attempted to develop a pattern of predation risk and then compare it to bird fledging patterns. In our study, despite the presence of a consistent predation activity pattern over the season and across the various grass habitats and the intensity of predation on fledglings in the immediate postfledging period (e.g., Cox et al., 2014; Naef‐Daenzer & Grüebler, 2016), evidence that the four species we studied actively avoided fledging during the period of high predation activity was low. Of the four species, one (Savannah Sparrow) fledged significantly more during the high predation activity period at both temporal scales. At the fine‐scale, increased fledging during the latter part of the morning period of low predation activity was common for all species but the species also fledged more than expected during the morning average predation activity period. Only one of the four species (Grasshopper Sparrow) fledged more than expected during the period of low predation activity at the coarse‐scale. Because Bobolink and Eastern Meadowlark had hours of increased fledging throughout the day (fine‐scale), Bobolink and Eastern Meadowlark fledged at random with respect to the predation activity periods (coarse‐scale). Overall, while the species fledged significantly more during certain hours across the morning, there was little evidence that predation risk was a driver affecting the species' fledging patterns.

Although Ribic, Ng, et al. (2018), and Ribic et al. (2019) suggested that nestling energetics were a primary driver for the timing of fledging for grassland birds, those studies lacked the context of the predator activity cycle. We now have that context. From this study, we found that fledging earlier in the morning had the ancillary benefit that nestlings left the nest during a period when predation activity was low relative to the rest of the diel predation cycle (i.e., reduced risk of an immediate encounter with a predator). However, a substantial fraction of nestlings fledged outside of the early morning low predation activity period as the nestlings balanced the relative costs and benefits of leaving the nest throughout the day. The idea that energetics plays a role in the timing of fledging has been a common alternative to predation as the driver in studies outside of the grassland system (Chiavacci et al., 2015; Johnson et al., 2004; Santema et al., 2021).

Relative to the species we studied, there is an interesting question of whether Grasshopper Sparrow's greater use of the low predation risk period was a side effect of starting the day earlier than the other species or an intentional predation risk avoidance strategy. If predation risk avoidance is a strategy for when nestlings fledge, nestlings that fledge earlier in the day should experience higher survival during the fledgling period. While there has been relatively little work done on this subject, two recent studies directly assessed this question for two species of cavity‐nesting birds. Radersma et al. (2015) found that Great Tits (*Parus major*) fledging earlier in the day led to higher recruitment to the breeding population the following year. In contrast, Santema et al. (2021) found that in Blue Tits (*Cyanistes caeruleus*) fledging earlier in the day did not have improved survival to the fall season. Much remains to be investigated regarding the potential linkage of time of fledging and fledgling survival.

While we focused on direct effects of predation, there may be indirect ways that predation affects species differences in fledging time, though studies are lacking on this topic. Some insight may come from studies on the dawn chorus. Recent work on timing of the dawn chorus in forest systems (Netteland, 2018; Ulltang, 2018) implicated predation avoidance as a driver of when species start singing, such that species started their dawn chorus when their visual capabilities enabled them to detect avian predators (Netteland, 2018). In the grassland system, an indirect effect of predation may manifest itself in relation to time of fledging by affecting when adults provision their young. Santema et al. (2021) argued that the time of day that nestlings fledged was linked to the nestlings being energetically ready. If grassland bird species vary when adults begin their daytime activities (i.e., see their environment and detect predators) due to variation in visual capabilities, then nestlings of the different species will become energetically ready to fledge at different times, leading to a continuum of species' times of first fledging. We were not able to find any information on the visual capabilities of grassland bird species. However, Slay et al. (2012) reported that Grasshopper Sparrow adults started the day earliest before sunrise, Bobolink closer to sunrise, and Eastern Meadowlark the latest after sunrise. These three species had the same order in fledging times with Grasshopper Sparrow fledging the earliest, followed by Bobolink, and then Eastern Meadowlark (Ribic, Ng, et al., 2018; this study).

Overall, as more researchers investigate processes that might affect the time of day that nestlings fledge in a variety of bird species (Chiavacci et al., 2015; Johnson et al., 2004; Lemel, 1989; Nilsson, 1990; Ribic, Ng, et al., 2018; Santema et al., 2021; Schlicht et al., 2012), it is becoming clearer that there are no universal answers—no single set of drivers appears to apply to all birds. As our technical capabilities to study this phase of fledging behavior improve, researchers will be able to address the complex interplay of nestling development, energetics, predation, and potentially other factors driving fledging behavior.

## CONFLICT OF INTEREST

None declared.

## AUTHOR CONTRIBUTIONS

**Christine A. Ribic:** Conceptualization (equal); formal analysis (equal); funding acquisition (equal); investigation (equal); methodology (equal); visualization (equal); writing–original draft (equal). **David Rugg:** Conceptualization (equal); data curation (equal); formal analysis (equal); methodology (equal); visualization (equal); writing–original draft (equal). **Kevin Ellison:** Investigation (equal); visualization (equal); writing–review and editing (equal). **Nicola Koper:** Visualization (equal); writing–review and editing (equal). **Pamela Pietz:** Funding acquisition (equal); investigation (equal); writing–review and editing (equal).

## Data Availability

The data analyzed in the paper (nest depredation and fledging time data categorized by hour before and after sunrise) are in Appendix 3. The base data files for the predation and fledging times are available from the Forest Service Research Data Archive (https://doi.org/10.2737/RDS‐2018‐0001‐2).

## APPENDIX 1

Number of grassland songbird nest depredations or visits by predator species in prairie and warm‐season and cool‐season Conservation Reserve Program fields. Data were collated from Pietz, Granfors, Grant, 2012, Ribic, Guzy, et al. 2012, Ellison et al. 2013, and Byers et al. 2017. All predation events where the predator could not be identified or where the camera was knocked over/askew were put into an Unknown group. Habitat and activity pattern information from Feldhamer et al. (2003) for mammals, Ernst and Ernst (2003) for reptiles, and Cornell Lab of Ornithology (2020) for birds. All scientific names were taken from the Integrated Taxonomic Information System (https://www.itis.gov). Species within the Predator group are ordered by number of depredations/visits.

**Table:**

Class	Predator group	Species	Activity pattern	Number of depredations/visits
Mammalia	Ground squirrels		Diurnal	
		Thirteen‐lined ground squirrel (*Ictidomys tridecemlineatus*)	Diurnal	116
		Franklin's ground squirrel (*Poliocitellus franklinii*)	Diurnal	5
	Meso‐mammals (grassland‐associated)		Diurnal/nocturnal	
		Striped skunk (*Mephitis mephitis*)	Crepuscular or nocturnal, emerging about sunset	32
		Least weasel (*Mustela nivalis*)	Diurnal/nocturnal	14
		American badger (*Taxidea taxus*)	Principally nocturnal; occasionally active during the day	12
		Red fox (*Vulpes vulpes*)	Nocturnal; peaks in activity common during crepuscular periods	3
		Coyote (*Canis latrans*)	Diurnal; tend to be more active during early morning and around sunset	2
		Unknown canid		1
	Meso‐ and large mammals (forest‐associated)		Crepuscular/nocturnal	
		White‐tailed deer (*Odocoileus virginianus*)	Crepuscular	14
		Raccoon (*Procyon lotor*)	Nocturnal	13
		Virginian opossum (*Didelphis virginiana*)	Nocturnal	6
	Mice/voles		Diurnal/nocturnal	
		Vole (*Microtus* spp.)	Diurnal/nocturnal	16
		Mouse (*Peromyscus* spp.)	Nocturnal	15
		Mouse or vole		2
	Other Mammals		Diurnal/nocturnal	
		Eastern cottontail (*Sylvilagus floridanus*)	Diurnal	2
		American mink (*Mustela vison*)	Primarily nocturnal	1
Reptilia	Snakes		Diurnal/crepuscular/nocturnal	
		Western foxsnake (*Pantherophis ramspotti*)	Primarily diurnal but may be active at night during warm rains	46
		Eastern milksnake (*Lampropeltis triangulum*)	Primarily nocturnal; may be active during daylight hours in spring and fall.	24
		Plains garter snake (*Thamnophis radix*)	Activity controlled by air temperature; diurnal, crepuscular, or nocturnal, depending on temperature (shift to nocturnal activity under hot temperatures)	6
		Snake spp.		6
Aves	Birds		Diurnal	
		Brown‐headed Cowbird (*Molothrus ater*)	Diurnal	12
		Eastern Meadowlark (*Sturnella magna*)	Diurnal	5
		Northern Harrier (*Circus cyaneus*)	Diurnal	4
		Red‐tailed Hawk (*Buteo jamaicensis*)/Buteo	Diurnal	5
		American Kestrel (*Falco sparverius*)	Diurnal	1
		Red‐winged Blackbird (female) (*Agelaius phoeniceus*)	Diurnal	1
		Savannah Sparrow (*Passerculus sandwichensis*)	Diurnal	1
	Unknown			21
	Unknown Mammals			6

## APPENDIX 2

Residuals from models of fledging pattern of 4 obligate grassland songbird species for first fledge events (First nestling) and subsequent nestling fledge events (Nestling 2+) against the relative predation activity pattern during the active period of the grassland bird species (civil twilight (hour −1) through sunset (hour 15)). Local sunrise is hour 0. Bolded positive and negative residuals indicate fledging that occurred at higher or lower rates than predicted by the rates of hourly relative predation activity at *α* of 0.10. Species are ordered by average time of first fledging, earliest to latest.

**Table:**

Hour	Relative predation activity	Residuals							
		Grasshopper sparrow		Savannah sparrow		Bobolink		Eastern meadowlark	
		First nestling	Nestling 2+	First nestling	Nestling 2+	First nestling	Nestling 2+	First nestling	Nestling 2+
−1	Low	−0.7	−0.94	−0.74	−1.03	−1.15	−1.6	−1.09	**−1.46**
0	Low	−0.7	−0.94	0.61	**1.87**	−0.28	−1.6	−0.18	−0.78
1	Low	**7.83**	**7.57**	**3.32**	−0.06	**2.32**	0.28	**4.39**	−1.46
2	Low	**4.99**	**9.7**	**1.97**	**2.84**	**8.4**	**8.43**	**2.56**	**4.7**
3	Average	0.76	0.61	1.49	−0.34	**3.28**	**6.46**	**3.64**	**7.06**
4	Average	−0.16	**1.99**	0.61	1.54	**3.85**	**2**	**1.86**	1.29
5	High	0.3	**2.42**	**2.41**	**1.88**	−0.16	1.62	−0.46	**−2.26**
6	High	0.32	−1.1	−0.58	−0.24	−0.14	1.66	−0.95	−0.34
7	High	−1.26	**−1.69**	−1.33	**1.92**	−1.59	**−2.17**	0.07	0.04
8	High	−1.27	**−1.7**	−0.59	**−1.87**	−1.6	**−2.19**	−1.47	−0.37
9	Average	−1.09	−1.45	−1.14	−0.34	−0.09	−0.84	−0.5	−1.81
10	Average	−1.09	−1.45	−1.14	−0.34	−1.78	−0.03	**−1.69**	**−2.25**
11	Average	−1.09	−1.45	−1.14	−1.59	−0.65	−0.84	1.27	1.29
12	Average	−1.09	−1.45	−0.26	−0.97	−1.22	−1.65	−0.5	1.29
13	Average	−0.16	−1.45	−1.14	−0.34	**−1.78**	**−2.46**	−1.1	−1.81
14	Average	−0.16	−1.45	−0.26	−0.34	**−1.78**	**−2.46**	**−1.69**	−0.48
15	Average	−1.09	−1.45	−0.26	−1.59	**−1.78**	**−2.46**	**−1.69**	−1.81

## APPENDIX 3

Grassland songbird nest depredations in prairie and warm‐season and cool‐season Conservation Reserve Program fields and fledging events of 4 obligate grassland songbird species categorized by hour before and after sunrise. Fledging events were divided into first fledge events (First nestling) and subsequent nestling fledge events (Nestling 2+).

**Table:**

Hour before/after sunrise	Nest depredations	Grasshopper Sparrow		Savannah Sparrow		Bobolink		Eastern Meadowlark	
		First nestling	Nestling 2+	First nestling	Nestling 2+	First nestling	Nestling 2+	First nestling	Nestling 2+
3–4 hr before	12	0	0	0	0	0	0	0	0
2–3 hr before	7	0	0	0	0	0	0	0	0
1–2 hr before	9	0	0	0	0	0	0	0	0
0–1 hr before	15	0	0	0	0	0	0	0	0
0–1 hr after	5	0	0	1	3	1	0	1	1
1–2 hr after	6	6	8	3	1	4	3	6	0
2–3 hr after	6	4	10	2	4	11	16	4	9
3–4 hr after	23	2	3	3	2	9	22	9	21
4–5 hr after	23	1	5	2	5	10	11	6	8
5–6 hr after	28	2	7	5	7	4	13	3	1
6–7 hr after	21	2	1	1	3	4	13	2	6
7–8 hr after	26	0	0	0	7	1	2	4	7
8–9 hr after	27	0	0	1	0	1	2	1	6
9–10 hr after	22	0	0	0	2	3	4	2	1
10–11 hr after	22	0	0	0	2	0	6	0	0
11–12 hr after	13	0	0	0	0	2	4	5	8
12–13 hr after	23	0	0	1	1	1	2	2	8
13–14 hr after	10	1	0	0	2	0	0	1	1
14–15 hr after	18	1	0	1	2	0	0	0	4
15–16 hr after	14	0	0	1	0	0	0	0	1
16–17 hr after	19	0	0	0	0	0	0	0	0
17–18 hr after	11	0	0	0	0	0	0	0	0
18–19 hr after	21	0	0	0	0	0	0	0	0
19–20 hr after	11	0	0	0	0	0	0	0	0
